# Pilot study shows skin-to-skin care with parents improves heart rate variability in preterm infants in the neonatal intensive care unit

**DOI:** 10.3389/fped.2023.1269405

**Published:** 2023-09-18

**Authors:** Erin Swieter, Jessica M. Gross, Julia Stephen, Kristi Watterberg, Jessie R. Maxwell

**Affiliations:** ^1^ Department of Pediatrics, University of New Mexico, Albuquerque, NM, United States; ^2^ Clinical and Translational Science Center, University of New Mexico, Albuquerque, NM, United States; ^3^ The Mind Research Network a Division of Lovelace Biomedical Research Institute, Albuquerque, NM, United States; ^4^ Department of Neurosciences, University of New Mexico, Albuquerque, NM, United States

**Keywords:** heart rate variability, skin-to-skin, preterm infants, intubation, kangaroo care

## Abstract

**Background:**

Skin-to-skin care in the newborn intensive care unit typically lasts for short periods of time and enhances breastfeeding, attachment, and parental self-esteem. Heart rate variability (HRV) increases with gestational age and is a measure of maturation of parasympathetic vs. sympathetic autonomic nervous system activity. HRV measurements may be useful in capturing changes in autonomic regulation in response to skin-to-skin care.

**Objective:**

To analyze the effects of skin-to-skin care on HRV in preterm infants receiving respiratory support. We hypothesized that skin-to-skin care would result in a more mature pattern of parasympathetic activity.

**Methods:**

In this prospective crossover study, infants <30 weeks' gestation and 1–6 weeks postnatal age had HRV recorded for 30 min before, during, and after skin-to-skin care sessions. HRV characteristics analyzed included the standard deviation of the normal-to-normal interval (SDNN), the root mean squared of successive differences of normal-to-normal intervals (RMSSD), and the standard deviation of decelerations (SDDec).

**Results:**

10 infants between 25 5/7–29 6/7 weeks gestational age and 7–41 days postnatal age completed 22 sessions while receiving respiratory support (positive pressure ventilation or nasal cannula oxygen). Two measures of HRV (SDNN and RMSSD) were significantly decreased by the end of the skin-to-skin sessions, compared to pre-session values. SDNN decreased from a median of 10.44 ms before the session to 6.70 ms after being placed back in bed (*p* < 0.05), with RMSSD decreasing from a median of 6.80 ms before the session to 4.32 ms while being held at the end of 30 min (*p* < 0.05).

**Discussion:**

Skin-to-skin care with a parent resulted in a more mature autonomic nervous system pattern in preterm infants receiving respiratory support, suggesting physiologic benefit for the infant. No adverse events were seen during any session.

## Introduction

Skin-to-skin care for newborns was originally implemented in low resource countries as Kangaroo Mother Care (KMC), with preterm newborns on their mother's chest 24 h/day. KMC has been shown to be effective for thermal control, breastfeeding, and bonding in newborns (1). For infants with a birthweight <2,000 g born in low- or middle-income countries, initiating KMC within the first week of postnatal life resulted in 51% reduction in mortality (1). KMC has also been found to decrease health care related sepsis and to improve infant growth (1).

In contrast, skin-to-skin care in neonatal intensive care units (NICUs) in high-resource countries is usually performed as short-term placement on mother's or father's chest to enhance breastfeeding, attachment, and parental self-esteem. Studies have shown that it is safe for both intubated and non-intubated preterm infants (1–3). However, a survey found that 59% of NICU nurse managers thought intubated infants should not receive skin-to-skin care for reasons including lack of criteria for infant selection, fear of extubation, stress to the infant, time involved for the nurse, temperature control, and bedside nursing fears of being blamed if something went wrong (4). Demonstration of potential benefit of skin-to-skin care for the infant could promote expansion of this practice. One potential benefit could be enhancement of the maturation of the autonomic nervous system.

The autonomic nervous system, comprised of sympathetic and parasympathetic innervations, is incomplete at birth. In premature infants (<37 weeks' gestation), sympathetic tone is dominant. Increased parasympathetic tone promotes growth and energy conservation (5), maintenance of appropriate muscle tone in the alimentary canal and decreased heart rate, thus allowing cardiac muscle rest and building up reserves for times of stress (6). Heart rate variability (HRV) is the temporal variation between sequences of consecutive heart beats, measured by the normal to normal (NN) interval, which is the period between adjacent QRS complexes (7). The standard deviation of the normal-to-normal interval (SDNN) is a metric of HRV that represents the overall heart rate variability, while the root mean squared of successive differences of normal-to-normal intervals (RMSSD) evaluates variation in adjacent N-N intervals to give an estimate of changes in short-term HRV (7). Heart rate variability measures the balance between sympathetic and parasympathetic mediators of heart rate (5). Variability increases with gestational age and with skin-to-skin care (8–10). There are few published studies reporting HRV in preterm infants receiving respiratory support, although one study of HRV in 11 preterm infants showed a statistically significant change in six of eight HRV measures (10). Thus, HRV measurements may be useful in capturing clinically relevant dynamic changes in autonomic regulation in response to skin-to-skin care (9–11).

Additionally, there are few studies of skin-to-skin care with fathers; however, this care has been shown to contribute to the father's development of positive feelings of attachment and bonding (12). Results of a crossover trial comparing physiologic measures in preterm infants before, during, and after skin-to-skin care showed no significant difference in body temperature, heart rate, respiratory rate, or neonatal stress score between care by the infant's mother or father, although oxygen saturations were higher during skin-to-skin with mothers (13).

The objective of this pilot study was to examine heart rate variability in preterm infants receiving respiratory support during skin-to-skin care with mothers and fathers. We hypothesized that skin-to-skin care would be associated with a more mature pattern of parasympathetic activity as measured by various domains of heart rate variability. An exploratory aim was to investigate differences in infant heart rate variability between skin-to-skin care performed by mother or father.

## Materials and methods

**Figure 1:**
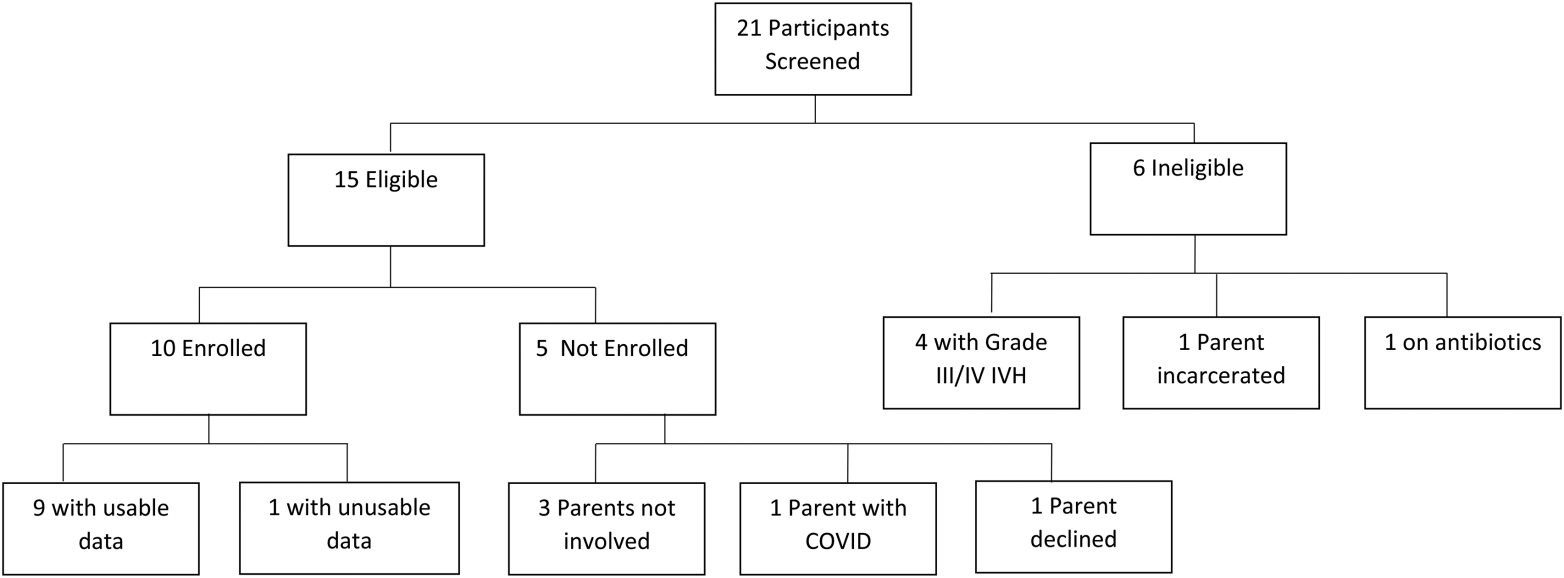
Participant screening and enrollment.

This prospective pilot study was approved by the Institutional Review Board at the University of New Mexico Health Sciences Center, and written parental consent was obtained for all participants. Infants were eligible for the study if they were born at ≤30 weeks’ gestational age, were less than 6 weeks postnatal age, were without Grade III or IV intraventricular hemorrhage (IVH) on cranial ultrasound (14), and were receiving some degree of respiratory support at the time of the first session. This could include mechanical ventilation, continuous positive airway pressure, high or low flow nasal cannula support. Infants were excluded if they had a known genetic disorder or chromosomal anomaly, major congenital anomaly, were undergoing active sepsis evaluation or treatment for infection, or receiving blood pressure or cardiac medications. Infants meeting study criteria were approached for consent, and consented infants were eligible to participate in a maximum of six skin-to-skin sessions (Figure 1).

The study sessions consisted of a 30-min observation period prior to the skin-to-skin care, a skin-to-skin care session lasting at least 30-min, and a 30-min observation period after completion of skin-to-skin care, as described below. During the skin-to-skin epoch, each infant was positioned upright on the parent's chest, with the infant's head turned towards one side. Heart rate variability data were collected by placing three electrocardiogram (ECG) leads and a pneumogram on the infant and was recorded using the BioPac Systems MP150 hardware (California, USA), the DA100C (pneumogram), the ECG100c (hardwired) equipment, and Acqknowledge 4.4 software. Initially a wireless system was used to capture data; however, interference from other NICU equipment necessitated switching to a hardwired system. All data presented in this manuscript were acquired with the hardwired system. HRV sensors and the pneumogram were removed following data collection completion, using massage oil to reduce skin irritation.

The SDNN, the RMSSD, and standard deviation of deceleration (SDDec) were used as the measures of heart rate variability (HRV). Both SDNN and RMSSD are time-domain measures, with SDNN reflecting parasympathetically-mediated changes and RMSSD reflecting vagally mediated changes in the HRV (15). SDNN provides insight into the overall heart rate variability, while RMSSD reflects short-term variation from one beat to the next (16). SDDec was calculated as it measures the standard deviation of all NN-intervals, and thus can provide information on regulatory instability (16). QRSTool was used to clean the HRV data and obtain 5 min segments of data from the following timepoints relative to the 30 min STS session: 5 min before starting skin-to-skin (pre skin-to-skin), the first 5 min of the skin-to-skin session (during, beginning of skin-to-skin), the last 5 min of the 30 min skin-to-skin session (during, end of skin-to-skin), and the first 5 min once the infant was placed back into the bed (post skin-to-skin session). The 30-min skin-to-skin session time was chosen as it is challenging for many caregivers to be present for multiple hours at the bedside. Additionally, while other studies have used longer periods, any positive changes observed in 30 min would support skin-to-skin when possible. Kubios HRV Scientific was used to obtain SDNN and RMSSD from the interbeat interval (IBI) obtained from QRSTool, and SDDec was calculated from the RR intervals given by Kubios using the equation from O. Nasario-Junior (17).

## Statistical analysis

Measures of HRV were taken across the pre, during, and post skin-to-skin sessions. SDNN, RMSSD, and SDDec were analyzed separately, using Friedman's tests followed by *post-hoc* Wilcoxon signed-rank tests for significant results. We analyzed these measures for 5 min at the beginning of the skin-to-skin contact session, then repeated the analysis with 5 min during the end portion of the skin-to-skin session to determine whether the effect of the contact on HRV changed over the course of the session. Skin-to-skin session HRV measures were then compared to post-session measures to evaluate persistence of any changes. Prior to analysis, the distributions of HRV measures within the two groups at each time point were tested for normality using Shapiro–Wilk tests. None of the HRV measures were normally distributed within groups according to these tests, so Mann–Whitney *U*-tests were used to evaluate differences between groups. We also tested the hypothesis that there would be a difference between sessions with mother and father. For this analysis, observations were separated into two groups according to which parent provided the skin-to-skin contact.

A *p*-value of 0.05 was used to determine significance for the analyses. All analyses were conducted in R 4.1.1 (R Foundation for Statistical Computing, Vienna, Austria).

## Results

Ten infants between 25 5/7–29 6/7 weeks gestational age and 7–41 days postnatal age completed 22 sessions while receiving respiratory support, either positive pressure ventilation or nasal cannula oxygen, with continuous positive airway pressure support being the most common (Table 1). One participant's data was unusable; therefore, 20 sessions from 9 participants were included in the analyses. All skin-to-skin sessions lasted more than 30 min. Table 2 shows participants' characteristics at birth, with the corrected age at the time of each session shown in Supplementary Table S1.

**Table 1:** Number of sessions completed on varying respiratory support.

Type of respiratory support	Number of sessions	% of total sessions
Low flow nasal cannula	2	9%
High flow nasal cannula	4	18%
Continuous positive airway pressure	14	64%
Nasal intermittent positive pressure ventilation	2	9%

**Table 2:** Participant characteristics.

		*N*	%
Sex	Female	4	44
	Male	5	56
Gestational age (weeks)	25	1	11.1
	26	0	0
	27	2	22.2
	28	2	22.2
	29	4	44.4
GA median (25–75%ile)	29 (28.2–29.2)		
Birthweight median (25%–75%ile)	1,127 (898–1,400)		
Parent completing session	Mothers	14	70
	Fathers	6	30

GA, gestational age.

SDNN and RMSSD were significantly decreased between the pre-skin-to-skin data and the last 5 min of the skin-to-skin session, reflecting a more mature pattern. As anticipated, there was no difference between pre-session and the first 5 min of the skin-to-skin session (Table 3). The *post-hoc* tests showed significant differences between pre-skin-to-skin care and during the session for both SDNN and RMSSD, and a significant difference between the during- and post- skin-to-skin session for RMSSD (Table 4). There was no significant difference in these measures prior to skin-to-skin contact with either parent, ruling out any underlying bias.

**Table 3:** Results using Friedman's test for comparing three domains of HRV before, during and after skin-to-skin care; additionally showing the beginning of skin-to-skin compared to the end of skin-to-skin sessions.

HRV measure	Sessions	*p*-value	Adjusted *p*-value	Effect size (Kendall's *W*)
SDNN	Pre, during (beginning), post	0.06	0.12	
	Pre, during, post	0.049^a^	0.12	0.333 (moderate)
RMSSD	Pre, during (beginning), post	0.37	0.55	
	Pre, during, post	0.01^a^	0.08	0.481 (moderate)
SDDec (in seconds)	Pre, during (beginning), post	0.46	0.55	
	Pre, during, post	0.72	0.72	

HRV, heart rate variability; SDNN, standard deviation of the normal-to-normal interval; RMSSD, root mean squared of successive differences of normal-to-normal intervals; SDDec, standard deviation of decelerations.

^a^ Signifies statistical significance with *p* < 0.05.

**Table 4:** SDNN and RMSSD *post-hoc* Wilcoxon signed-rank tests comparing pre skin-to-skin with post skin-to-skin, pre skin-to-skin with the end of skin-to-skin, and the end of skin-to-skin with post skin-to-skin.

SDNN					
Timepoint 1	Timepoint 2	Statistic	*p*-value	Adjusted *p-*value	Adjusted *p* significance
Pre	Post	41	0.027	0.041	^a^
Pre	During	42	0.02	0.041	^a^
Post	During	30	0.426	0.426	ns
RMSSD					
Timepoint 1	Timepoint 2	Statistic	*p-*value	Adjusted *p-*value	Adjusted *p* significance
Pre	Post	37	0.098	0.098	ns
Pre	During	44	0.008	0.018	^a^
Post	During	43	0.012	0.018	^a^

SDNN, standard deviation of the normal-to-normal interval; RMSSD, root mean squared of successive differences of normal-to-normal intervals.

^a^ Signifies statistical significance with *p* < 0.05; all groups had nine participants’ sessions analyzed.

The Mann–Whitney *U*-tests showed no statistically significant difference in infant HRV measures between sessions with fathers and mothers at any time point, though the limited sample size does not allow for assessment of heart rate variability from skin-to-skin between mothers and fathers Table 5). Table 6 shows the median and interquartile range for three HRV measures at each time point for the full sample.

**Table 5:** Results of the Mann–Whitney *U*-tests comparing mothers and fathers at each timepoint.

HRV measure	Skin-to-skin session	Mother median (IQR)	Father median (IQR)	*p*-value
SDNN	Pre	11.54 (17.34)	6.22 (5.80)	0.56
	During (beginning 5 min)	9.71 (2.15)	7.06 (2.40)	0.56
	During (end 5 min)	6.11 (4.93)	5.38 (6.31)	0.90
	Post	9.68 (8.23)	6.35 (4.69)	0.73
RMSSD	Pre	5.18 (5.16)	9.83 (6.86)	0.41
	During (beginning 5 min)	4.66 (3.47)	5.13 (0.61)	0.73
	During (end 5 min)	4.43 (2.08)	3.72 (2.10)	1.00
	Post	7.59 (5.06)	5.24 (2.25)	0.41
SDDec	Pre	0.009 (0.011)	0.006 (0.009)	0.56
	During (beginning 5 min)	0.011 (0.008)	0.002 (0.004)	0.19
	During (end 5 min)	0.002 (0.008)	0.004 (0.007)	0.73
	Post	0.006 (0.007)	0.011 (0.010)	0.73

HRV, heart rate variability; SDNN, standard deviation of the normal-to-normal interval; RMSSD, root mean squared of successive differences of normal-to-normal intervals; SDDec, standard deviation of decelerations.

**Table 6:** HRV measure at each time point for full sample.

HRV measure	Skin-to-skin session	Median (IQR)
SDNN	Pre	10.44 (7.82)
	During (beginning 5 min)	7.58 (3.29)
	During (end 5 min)	6.11 (3.16)
	Post	6.70 (6.40)
RMSSD	Pre	6.80 (5.84)
	During (beginning 5 min)	4.66 (2.02)
	During (end 5 min)	4.32 (1.57)
	Post	4.76 (3.10)
SDDec	Pre	0.009 (0.007)
	During (beginning 5 min)	0.009 (0.007)
	During (end 5 min)	0.008 (0.007)
	Post	0.010 (0.007)

HRV, heart rate variability; SDNN, standard deviation of the normal-to-normal interval; RMSSD, root mean squared of successive differences of normal-to-normal intervals; SDDec, standard deviation of decelerations.

## Discussion

In this pilot study, the effects of skin-to-skin on three variables of HRV (SDNN, RMSSD, and SDDec) were analyzed during 20 skin-to-skin sessions in nine preterm infants. We assessed the preterm infant's autonomic nervous system regulation during skin-to-skin with both mothers and fathers. We found that at the end of a 30-min skin-to-skin session, SDNN and RMSSD showed a significantly more mature autonomic nervous system pattern, indicating a change from a predominantly sympathetic state to predominantly parasympathetic activity (6). The change observed in SDNN persisted for at least 10 min after the infant was placed back in his/her bed, suggesting continuation of the effect. Interestingly, these physiologic changes may also directly impact cerebral blood flow. As noted by Sehgal et al., significant improvements in cerebral blood flow occur in response to skin-to-skin care (18). This highlights the importance of encouraging skin-to-skin care with caregivers whenever possible, as the impact could have significant and long-lasting effects.

All infants maintained normothermia, and no session was terminated for significant bradycardia, indicating that skin-to-skin care as performed in this study was well tolerated (3, 10). As recommended by the American Academy of Pediatrics, infants in the NICU should be on continuous cardiac monitors, maintain airway patency with appropriate head positioning, and ensure endotracheal tubes, arterial and venous lines and other life support equipment is stable during skin-to-skin care (1).

Only one previous study looked at HRV with fathers; however, infants in that study were more mature, between 30 and 36.6 weeks' gestation (19). Although no statistically significant differences were found between mothers and fathers in this study, the small sample size included in the study does not allow us to assess whether there are differences in heart rate variability when the skin-to-skin contact is performed by the mother or the father. Our results indicate that preterm infants less than 30 weeks' gestation can physiologically benefit from skin-to-skin care with both parents, and we hope that these results encourage providers to have fathers do skin-to-skin care, not only for the development of bonding, but also for increased parasympathetic activity. It is not uncommon for fathers to be the first parent to hold their preterm infant in the NICU, depending on the mother's clinical status.

Limitations of this study included, first, a small sample size of 9 babies; however, even with this limited sample size we observed beneficial changes in the HRV measures monitored, which supports a large effect size and may encourage further exploration of the effects of skin-to-skin care. Second, although our goal was to examine HRV in preterm infants requiring respiratory support, including intubated infants, no infant was receiving invasive mechanical ventilation at the time of skin-to-skin sessions. Third, the postnatal day of life during which skin-to-skin was completed varied, which can affect heart rate variability. Similarly, certain medical conditions such as a patent ductus arteriosus may also impact the heart rate variability, but was not collected as it was beyond the scope of this project. Additionally, we did not have the resources to analyze the entire HRV session and were therefore unable to determine whether the effects of skin-to-skin lasted longer than 10 min after the end of a session. Lastly, we did not require parents to terminate skin-to-skin after the 30 min; therefore, some infants continued a session for up to 120 min, and we did not analyze how the duration of skin-to-skin care may have affected the results. Strengths of this study include the design, where each infant acted as his/her own control, the inclusion of extremely preterm infants receiving respiratory support, and the inclusion of sessions with fathers.

In conclusion, our study supports previous studies that found skin-to-skin care to be safe for preterm infants. In addition, we showed that such sessions can lead to a more mature autonomic nervous system pattern, as measured by heart rate variability, and that this benefit was similar for fathers and mothers.

## Data Availability

The raw data supporting the conclusions of this article will be made available by the authors, without undue reservation.
