# Supplementary figures and images for: Pilot study shows skin-to-skin care with parents improves heart rate variability in preterm infants in the neonatal intensive care unit
Source: Front Pediatr. 2023 Sep 18;11:1269405. doi: 10.3389/fped.2023.1269405 (PMC10544889; doi:10.3389/fped.2023.1269405)

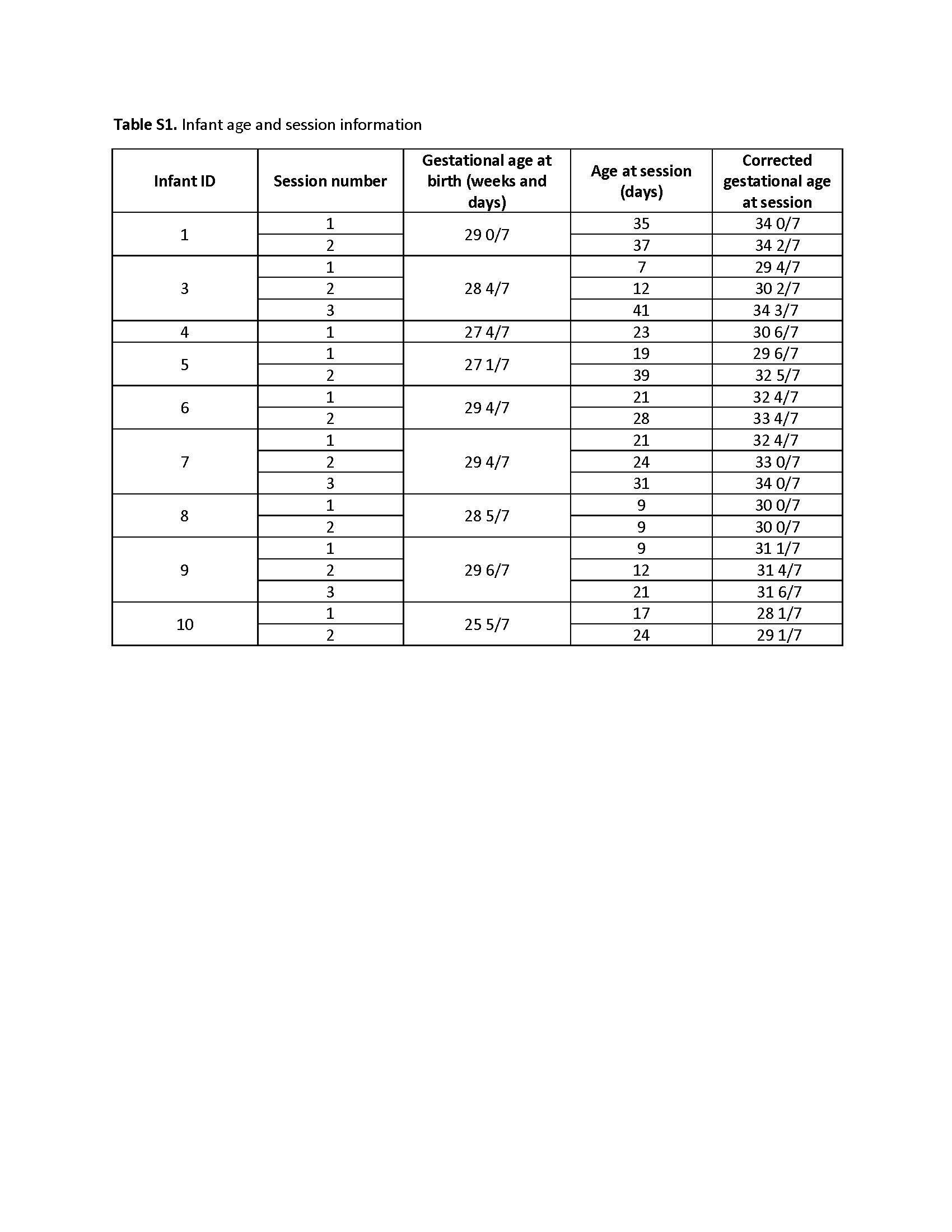

Supplement: Supplementary file 1 [file Image1.jpeg]
